# Designing Effective Hybrid Course Curriculum: A Design Science Approach to Gamification and Student Outcomes Validation

**DOI:** 10.1177/0193841X241291752

**Published:** 2024-10-09

**Authors:** An Duong Thi Binh, Thu-Hang Hoang, Huy Truong Quang

**Affiliations:** 1 CIRTech Institute, 638418 HUTECH University, Ho Chi Minh City, Vietnam; 2 School of International Business – Marketing, 145467 University of Economics Ho Chi Minh City, Ho Chi Minh City, Vietnam; 3 The Business School, 113073 RMIT International University, Ho Chi Minh City, Vietnam

**Keywords:** gamification, hybrid learning, student engagement, satisfaction, design science, Vietnam

## Abstract

In the modern educational landscape, the integration of gamification into hybrid learning environments has emerged as a promising approach to enhance student outcomes. However, there remains a lack of comprehensive frameworks for designing gamified hybrid courses and validating their impact on student outcomes. This paper proposes a design science-based approach to gamified course design in hybrid learning contexts. Drawing on the principles of design science research, we developed a framework for designing a gamified hybrid course curriculum that incorporates course content, activities, and assessments based on four elements of gamification (achievement elements, utilitarian value, hedonic benefits, and competition). To validate the effectiveness of our approach, we conducted a study with 294 students enrolled in a hybrid business course that implemented the proposed gamification framework. Our findings indicate that all gamification elements of our proposed gamified hybrid courses positively enhance student engagement, achievement, and satisfaction. Ultimately, this paper not only contributes to the ‘gamification in education’ literature by providing a comprehensive framework for designing engaging and effective hybrid courses but also proposes a roadmap for the application of design science to embed gamification in business course curriculum design within the context of modern hybrid learning environments.

## Highlight

• This study provides a new way to design the course by applying design science to solve the learning and teaching problems in the business course.
• The relationship between the gamification element and student engagement was studied to show an impact on the behaviour and learning outcomes of students in higher education.
• The study’s findings indicate that the four gamification elements have a positive impact on student engagement in a hybrid learning model and indirectly influence student academic achievements and satisfaction.
• The course’s learning roadmap proposed in this study will address the gap between curriculum design and student engagement by creating a student performance-tracking dashboard.

## Introduction

After a prolonged period of being affected by the COVID-19 epidemic, while online learning offers flexibility and accessibility, it also presents challenges such as reduced student engagement and motivation compared to face-to-face instruction (Al-Hafdi & Alhalafawy, 2024). Even before and after the COVID-19 pandemic, research indicated that a significant percentage of students lacked concentration or interest in online learning (Gavriluță et al., 2022). These challenges persist beyond the pandemic, highlighting the ongoing need for innovative teaching methods that can combine remote and in-person learning to enhance student engagement and motivation (Nichols et al., 2022; Servant-Miklos, 2022; Tolppanen et al., 2022), which is called ‘hybrid learning’. *Hybrid learning* is a teaching and learning approach that combines in-person and virtual learning via the use of hybrid classroom resources such as learning management systems (LMS), video conferencing, and asynchronous (self-paced) learning (DuBois et al., 2019; Merritt et al., 2022; Nichols et al., 2022). Universities, like other service firms worldwide, are rapidly embracing digitalisation, marking a significant shift from traditional to modern computing-based information and communication technologies (Hoang & Le, 2024). This transition is accompanied by the increasing adoption of hybrid learning techniques (Merritt et al., 2022; Nichols et al., 2022).

Simultaneously, the notions of gamification and game-based learning are two persisting tactical teaching trends due to their ability to facilitate a more intuitive and engaging learning experience, along with the growing popularity of esports and the constant inclusion of new classroom equipment (Al-Hafdi & Alhalafawy, 2024; Navarro-Espinosa et al., 2022). *Gamification of learning* is an educational approach that uses game aspects and design in both undergraduate and postgraduate learning settings to inspire students (van Gaalen et al., 2021). For example, Kahoot! embodies an innovative evolution of undergraduate student response systems that emphasises gamification to elevate student motivation and engagement (Wang et al., 2016). This platform is ideal for raising students’ interest, engagement, and satisfaction as well as evaluating their comprehension of a course (Lin et al., 2018). Moreover, gamification in postgraduate teaching has proved to be an effective tool to promote more student engagement, learner attrition, and knowledge retention (Nevin et al., 2014; Raju et al., 2021); enhance learning progression and social connectedness (Haque et al., 2017); and possibly create more motivation for learners (Navarro-Espinosa et al., 2022). Besides, gamification is becoming a valuable tool for educators because of the rising focus on student participation and the opportunities given by digital learning (Dunn & Kennedy, 2019). Compared to traditional classes, 67% of students found gamified learning more engaging and motivating (Chapman & Rich, 2018). Race and Powell (2000) also demonstrated the interplay between the good design of teaching activities and the cooperation of students in the learning process, reflected in the quality of learning. It is evident that gamification and game-based learning have become ‘trendy’ teaching strategies as they contribute to making undergraduate and postgraduate learning more intuitive and engaging (Chapman & Rich, 2018; Lin et al., 2018; Nevin et al., 2014; Oliveira et al., 2023), yet their effectiveness in various settings is still unclear, often resulting in unsatisfactory learning outcomes. For instance, gamification has mostly been studied in in-person or virtual learning environments (Oliveira et al., 2023; Sanchez et al., 2020), overlooking hybrid learning’s particularities (O’Byrne & Pytash, 2015). Due to its flexibility and global reach, hybrid courses in higher education have gained better ground (Raes et al., 2020; Singh et al., 2021). However, actively incorporating students in such contexts remains difficult. In conventional settings, gamification can boost student motivation and engagement (Raju et al., 2021), but its full potential in hybrid courses has yet to be confirmed empirically.

The objective of this study is to fill this gap by investigating the effects of incorporating gamification elements, such as competition, utilitarian value, achievement elements, and hedonic benefits, on student engagement, academic achievement, and satisfaction that hope to bring similar successes in the context of hybrid learning. Moreover, we propose to develop a suitable ‘roadmap’ model for the implementation of gamification in a higher education setting using design science theory. Design science (DS) has gained popularity as a research paradigm in the pedagogical realm, offering a systematic approach to creating and evaluating innovative educational artifacts for known problems (Antunes et al., 2021; Hevner et al., 2004; Thuan et al., 2019; Vom Brocke et al., 2020). Nevertheless, there is very limited research yet on applying the design science approach to improve the gamification experience of students, especially in hybrid learning settings (Leite et al., 2021). In this study, we aim to develop effective gamified hybrid learning environments by applying design science theory, which can improve the conventional curriculum through problem-solving and innovation (Henriksen et al., 2017). This will fill gaps in the literature and offer a novel approach to improving hybrid student engagement and learning outcomes in higher education. Current literature has supported that the ‘trendy’ pedagogical choice at all types of undergraduate, graduate, and PhD levels is DS education (Goldkuhl et al., 2017; Hevner, 2021; Winter & vom Brocke, 2021). We expect that this specific choice of teaching and learning approach is meant to help institutions and lecturers increase academic accomplishment and satisfaction among learners in hybrid learning environments by incorporating gamification into instructional design. Overall, the current investigation seeks to address three main research questions:

Question 1 What can be proposed as an alternative educational approach to embedding gamification in curriculum design for hybrid classes based on design science?

Question 2 When embedding gamification in curriculum design for hybrid classes, do gamification elements such as achievement elements, utilitarian value, hedonic benefits, and competition affect student engagement, and how do they affect it (negatively or positively)?

Question 3 When embedding gamification in curriculum design for hybrid classes, does student engagement positively affect academic achievement and student satisfaction in the hybrid learning context?

The main purpose of the study is to provide an explicit grasp of students’ interests in gamification elements, such as achievement elements, utilitarian value, hedonic benefits, and competition. The study additionally deals with the research questions of whether putting gamification into practice (i.e. in curriculum design for hybrid classes) will be effective through higher student satisfaction and academic achievement. Subsequently, universities and colleges can have a better understanding of their student’s needs and expectations in experiencing hybrid learning with gamification. Its implication points out to universities/colleges and lecturers whether gamification should be promisingly sustained. On that account, the suggestions supported by the research findings may guide the development and execution of gamified hybrid learning environments – ultimately augmenting the calibre of higher education.

This research adopts a case study approach, focussing on a specific university in Ho Chi Minh City, Vietnam. For confidentiality purposes, the institution will be referred to as “the University” throughout the paper.

## Literature Review

### Gamification

Gamification is the ‘use of game design features to inspire user behaviour in non-game environments’ (Deterding et al., 2011). Gamification, as per Domínguez et al. (2013), is the process of introducing game components into non-gaming software applications to improve user involvement, experience, and satisfaction. Gamification has been employed in myriad fields in recent years to improve employment outcomes in the development of their daily duties and jobs (Hugos, 2012; Pedreira et al., 2015). According to Gartner (2015), over half of the firms will enhance their user development procedures by 2015 since gamification provides immediate feedback, clear objectives, and tough activities. Gamification, as per Bíró (2014), has several characteristics of the theory of behaviourist learning, such as the supremacy of positive rewards, tiny step-by-step activities, instant feedback, and escalating difficulties. To affect learners’ behaviour, the utilisation of game-like rule structures, player experiences, and cultural roles are recommended in educational gamification (Su & Cheng, 2013).

Gamification is regarded as an essential novel approach to ensure both undergraduate and postgraduate student involvement and engagement not only in traditional face-to-face learning formats but also in new digital strategies such as fully online learning environments (Antonaci et al., 2019; Johnson et al., 2014; Kyewski & Krämer, 2018). Initially thought to be a strategy for increasing interest in education rather than improving the efficiency and effectiveness of the instruction (Reigeluth, 1999), gamification is anticipated to have direct effects on ‘individual learner performance’, which is the most crucial element in way-away learning approaches. This is clarified by its instinctive characteristics as a fun and effective approach to inspiring students in education and instruction (Lee & Hammer, 2011). The general objective of gamification in traditional face-to-face and fully online learning environments is to analyse the game aspects that make computer games interesting and to adapt and apply these features in educational-instructional processes to increase student involvement and loyalty to learning and educators to teaching activities (Antonaci et al., 2019; Simões et al., 2013). This element of entertainment helps users or students to concentrate on real-world issues by utilising the motivational capacity of computer games (Lee & Hammer, 2011). Gamification tactics to motivate and engage students have thrived in traditional learning contexts like classrooms and in-person encounters (Oliveira et al., 2023). Gamification has also been used in entirely online learning settings to encourage distant learners to participate and compensate for the loss of face-to-face interaction (Antonaci et al., 2019; Kyewski & Krämer, 2018). Gamification has the potential to lower dropout rates and poor individual motivation in remote education programs that use digital technology akin to e-learning (Sun & Rueda, 2012). Gamification, one of the most cutting-edge digital strategies (Johnson et al., 2014), can be used to achieve the set goals or integrated into the curriculum over a term (McFarland, 2017) and is a powerful contemporary technique to validate student engagement and commitment. The majority of participants in gamified learning activities are found to be undergraduate students (Al-Hafdi & Alhalafawy, 2024). Notably, although there have not yet been any studies investigating the differences in the effects of gamification on learners’ outcomes between undergraduate and postgraduate students, several mentions are worth consideration. For instance, Zhang et al. (2017) suggested that certain aspects they observed in their study, such as the usage of information and gamified activities, may be similarly appealing to all students, irrespective of their age or level of study; or a review study conducted by van Gaalen et al. (2020) concluded no clear evidence that the impact of gamification activities relied on contextual variables (e.g. undergraduate vs. postgraduate learning groups). On the other hand, Schlögl et al. (2021) noted that the anticipated results of gamified teaching and learning can differ based on the learners’ proficiency level, personal limitations, and preferences. Cheong et al. (2014) suggested that undergraduate students might be more likely to play games than postgraduate students) checked at the beginning of the educational course.

Gamification has shown clear benefits in traditional and fully online contexts, but its potential in hybrid classrooms remains largely untested. Hybrid classrooms, blending in-person and online learning, require tailored instructional design approaches (O’Byrne & Pytash, 2015). In this context, integrating gamification into hybrid course curricula is crucial for maintaining student engagement across both physical and virtual settings. Studies emphasise the importance of embedding gamification elements seamlessly into hybrid courses rather than using them merely as supplementary tools (Loos & Crosby, 2017; Sailer & Sailer, 2021). When thoughtfully integrated, gamification can significantly enhance student engagement, effectiveness, and satisfaction, fostering a state of flow in learning (Csikszentmihalyi, 1990). Simply adapting traditional teaching methods to online platforms may fail to fully engage students in hybrid learning (Ng & Lo, 2022, 2023). For example, a hybrid marketing class could be enriched by incorporating virtual quests, quizzes, and collaborative puzzles, transforming the learning experience into something immersive and dynamic (Bucchiarone, 2022). As hybrid classrooms and gamification gain traction in higher education (Antonaci et al., 2019), investigating their synergies is critical. Specifically, this research seeks to empirically confirm whether embedding gamification in hybrid learning can enhance student engagement, motivation, and outcomes, ultimately informing more effective instructional practices in today’s evolving educational landscape.

### Gamification in Curriculum Design

#### A DS Approach in Gamifying Hybrid Course Curriculum

**Figure 1.**
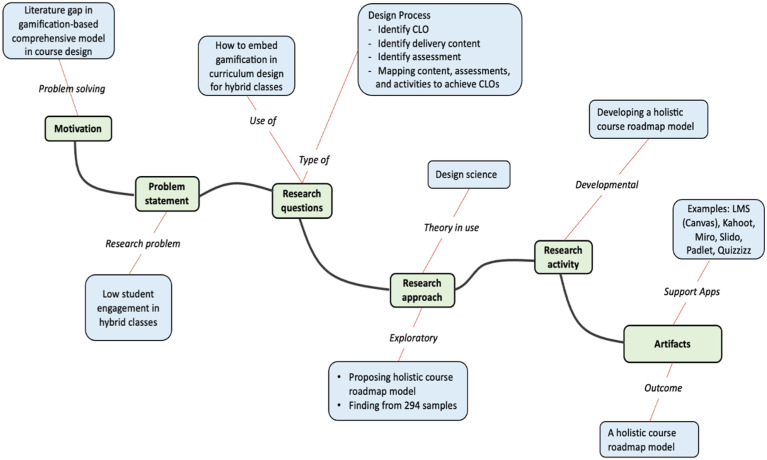
A DS analytical framework to implement gamification in curriculum development.

The seven elements of the DS framework include motivation, problem statement, research questions, research approach, theory-in-use, research activities, and research artifacts, which imply the steps to apply DS to solve a particular problem (Antunes et al., 2021). These elements are the base to create a modified framework used for this study (Figure 1).

Recognising the benefits of applying gamification to the curriculum design in the hybrid-teaching model, the research team was motivated to identify an appropriate approach that academic staff can refer to when designing a course’s curriculum. This study’s outcome also addresses the literature gap of lacking a comprehensive model to implement gamification to improve student engagement (Antonaci et al., 2019; Johnson et al., 2014; Kyewski & Krämer, 2018). The problem statement in DSR is useful for identifying the research problem of low student engagement in hybrid classes (Peffers et al., 2007).

The related DS research question in the study is ‘How to embed gamification in curriculum design for hybrid classes?’. The answer to this research question would address the problem of low student engagement in hybrid classes, and it was formulated by a design process of identifying the course’s learning outcomes (CLOs), delivery content, assessments, course activities, and the mapping and integration of these elements to achieve CLOs (Antunes et al., 2021; Henriksen et al., 2017). The exploratory research type is the approach for the study based on the DS implementation framework proposed by Thuan et al. (2019). Related to research activities, Hevner et al. (2004) suggested two main types: building and evaluating. In this study, we focused on building research activities to develop a holistic gamification-based learning roadmap that lists all learning content, assessments, and course activities in a single and simple map for better tracking and engagement. The artifact of the DS process is the holistic roadmap for the course’s curriculum design that incorporates course content, activities, and assessments (Thuan et al., 2019) based on four elements of gamification (achievement elements, utilitarian value, hedonic benefits, and competition) and various gamification-supporting applications such as learning management system (LMS – Canvas), Kahoot, Miro, and Slido.

#### Course’s learning roadmap

**Figure 2.**
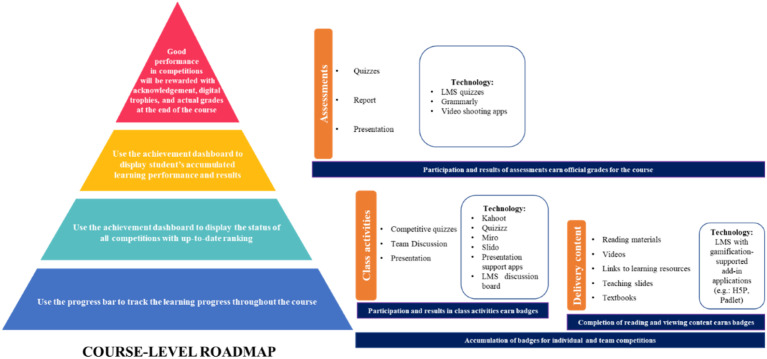
Explanation of the course-level learning roadmap in curriculum design.

The next step is identifying the answer to the first research question, ‘How to embed gamification in curriculum design for hybrid classes?’. By applying the DS implementation framework (Thuan et al., 2019) and student preferable gamification features such as progress bar, badges, ranking, rewards, and competition (Al-Hafdi & Alhalafawy, 2024), the research team proposed a holistic course-level learning roadmap in curriculum design, as illustrated in Figure 2.

One of the challenges in a hybrid learning model is that most interactions are spent with an LMS system that lays out the learning process in text-based document formats via Word or websites. This type of curriculum design lacks a big-picture view with attractive visualisation, limits tracking and communicating students’ learning progress, and causes inefficient engagement (Merritt et al., 2022). Thus, the course’s learning roadmap is proposed to address this gap. The roadmap is a simple, graphical, and one-page representation of the course’s learning content, class activities, and assessments designed to achieve the course’s learning outcomes. The roadmap will serve as a student performance tracking dashboard. Each item in the roadmap will include a link back to the source of information.

**Figure 3.**
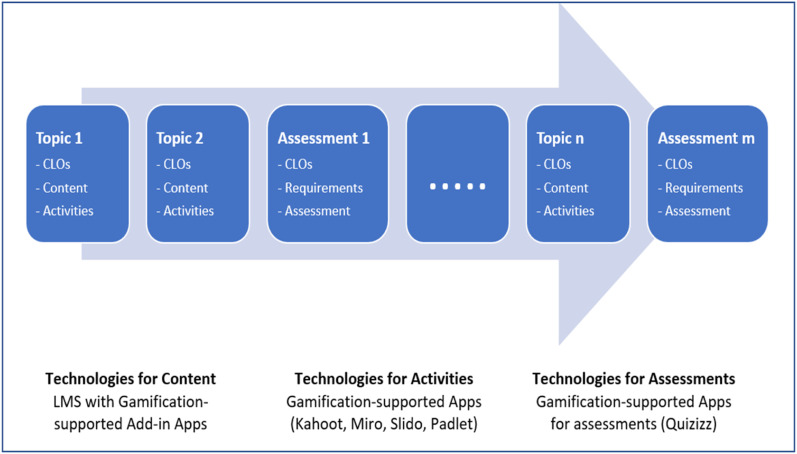
Gamification-based learning roadmap in curriculum design.

As schematically presented in Figure 3, there are three main elements in a course’s design: delivery content, class activities, and assessments, which often are divided into smaller components and offered weekly throughout the course’s learning period. The course’s delivery content includes reading materials, learning slides, visual videos, links to various learning resources, textbooks, and recommended articles and news. Class activities can be organised in various formats, such as competitive quizzes, team discussions, and presentations. Assessments can be conducted in quizzes, written reports, and presentations. Gamification features with all four elements of achievement elements, utilitarian value, hedonic benefits, and competition can be embedded in the three elements of a course’s design. When students complete their reading and view the required content, they can earn rewards such as badges, which are accumulated for various individual and team competitions. The roadmap can use a progress bar to show student learning progress throughout the course’s offering time. An achievement dashboard can be utilised to display the status of all competitions with up-to-date rankings. Students’ participation and results of class activities can earn badges and will be factored into competitions and displayed in the roadmap. Assessment results will earn official grades for the course and have different performance dashboards in the roadmap. Toward the end of the course, the results of the competitions can be converted into rewards of additional official grades to the course’s final grades. The conceptual proposal for the course’s level roadmap in curriculum design is summarised in Figure 3.

### Hypothesis Development

In this study, our goal is to confirm the positive outcomes of a gamified embedded hybrid learning experience including student engagement, motivation, and learning outcomes in hybrid mode. We believe that the Stimulus-Organism-Response (SOR) model (Mehrabian & Russell, 1974) offers an all-encompassing framework for comprehending the impact of gamification features on student behaviours and outcomes in hybrid classrooms. The model suggests that gamification features, which are *stimuli*, interact with specific internal processes of students (i.e. engagement), referred to as the *organism*, to produce certain outcomes such as academic achievement and satisfaction with learning (Robert & John, 1982). This approach allows us to explore the complex interplay between gamification elements, student characteristics, and learning outcomes within the unique context of hybrid classes.

Firstly, achievement elements are a version of gamification to encourage motivation and involvement in various systems (Hakulinen et al., 2015). Achievement elements, such as badges, trophies, stars, levels, and progress bars, are integral components of gamified learning environments that have been shown to positively impact student engagement (Ahmad et al., 2020). Research by Hakulinen et al. (2015) has found badges of achievement elements as a prominent method to motivate and foster students to learn. More specifically, by earning badges, trophies, or advancing through levels, students receive immediate feedback on their progress and accomplishments, which can increase their motivation to engage with course materials and activities and empower students to take ownership of their learning experiences (Van Ginkel et al., 2020). In hybrid learning, due to the lack of face-to-face interaction or in situations where students may face challenges in balancing their time between in-person and online activities, these elements serve as tangible rewards for their efforts, reinforcing their commitment to the learning process (O'Byrne and Pytash, 2015), and even allow them to showcase their accomplishments to their peers, fostering a sense of community and belonging (Mendoza & Venables, 2023). Hence, in the application of games in the hybrid educational environment, we believe that it is necessary to appreciate achievements to promote student learning and reinforce their learning ability.

H1 *Achievement Elements (AM) of a gamified hybrid course have a positive influence on Student Engagement.*

Next, we argue that utilitarian value (UV) is vital in boosting student engagement in gamified hybrid learning settings. UV is defined by its informational emphasis and its attention to the process of consuming, which leads to its association with efficacy and task-specific outcomes (Henry et al., 2004; Overby & Lee, 2006). In gamified hybrid courses, UV is attained by several means, including the provision of explicit learning objectives, constructive feedback on student’s progress, and tangible incentives for their accomplishments (Rios, 2021). For instance, students have the potential to earn assessment points or physical prizes when they finish game tasks, demonstrate proficiency in particular areas, or achieve specified milestones (Wolf, 2019). This feedback or achievement not only gives students a feeling of satisfaction but also improves their perception of the effectiveness of the learning experience (Yang et al., 2021). Research has also shown that UV directly affects student satisfaction and increased engagement (Aguiar-Castillo et al., 2019; Baptista & Oliveira, 2019). Thereby, we propose that:

H2 *Utilitarian value (UV) has a positive influence on Student Engagement.*

According to the perspective theory of flow, game challenges create hedonic benefits (Cowley et al., 2008; Csikszentmihalyi, 2014; Sweetser & Wyeth, 2005). Hedonic value is derived from visual appeal, entertainment, escapism, and enjoyment and can stem from having fun competing with others (Wolf, 2019). In game studies, social factors are proposed as predictors of players’ attitudes and intentions, and enjoyment is regarded as the most influential factor motivating gameplay (Akbari et al., 2021; Hsu & Lu, 2007). A similar study indicated that hedonic value would drive continued engagement intention (Högberg et al., 2019). Gamified learning environments greatly enhance student engagement through hedonic rewards, which include enjoyment, fun, and satisfaction (Ng & Lo, 2023). When it comes to gamified hybrid learning, such benefits are vital to motivate students involved in both in-person and online components of their coursework. More specifically, hedonic benefits increase students’ intrinsic motivation to engage with course materials and activities (Oluwajana et al., 2019). When hybrid learning is enjoyable and fun, students are more likely to be motivated to invest time and effort in their studies, leading to higher levels of engagement and active participation in both in-person and online learning activities. This intrinsic motivation has been demonstrated in research that enjoyment foresees the likelihood of using interactive (Akbari et al., 2021; Hamari, 2015; Moon & Kim, 2001; Van der Heijden, 2004; Venkatesh, 1999). Participants who perceive delight in games (gamification) are much more plausible to be prompted to play more (Bernecker & Ninaus, 2021; Hoon et al., 2002; Huang & Cappel, 2005; Yu & Huang, 2022). Moreover, in hybrid classes, particularly during designated online periods when students may experience isolation due to the absence of in-person interaction, the use of gamified learning environments (such as a Bamboozle or Gimkit group game to identify key theories or concepts of the topic) offers students a way to connect with their peers, exchange experiences, and collaborate on group projects (Antonaci et al., 2019; Kyewski & Krämer, 2018). This, in turn, enhances their engagement and sense of belonging, allowing them to actively participate in course activities.

We believe that by creating enjoyable and immersive learning experiences, gamified learning environments motivate students to actively participate in both in-person and online components of their course, leading to higher levels of engagement, motivation, and overall learning outcomes. As a result, we suggest the following hypothesis:

H3 *Hedonic Benefits (HBs) have a positive influence on Student Engagement.*

Competition persists as a predominant factor that incentivises students to partake in gamification tasks. In complex activities, a lack of skill can be compensated by competition. Competition elements, such as leaderboards, rankings, challenges, and rewards, considered one of the ten main qualities of successful game designs, could improve learning practices and outcomes as an aspect that might increase engagement (Reeves & Read, 2009; Vorderer et al., 2004). Competition elements, such as leaderboards, rankings, challenges, and awards, strongly impact student engagement in gamified hybrid learning environments (Yusof et al., 2021). Within the framework of hybrid learning, these factors are essential in fostering student motivation to actively engage in both the face-to-face and virtual aspects of their learning experience. Competition aspects provide a competitive atmosphere that drives kids to strive for excellence and surpass their classmates. Gamified hybrid learning environments enhance student participation and engagement by including leaderboards, rankings, and challenges. These features establish clear goals and objectives for students to strive toward, fostering a sense of success and appreciation for their efforts (Yusof et al., 2021). Through their presence on leaderboards, attainment of high ranks, and success in challenges, students gain social acknowledgement and validation for their achievements, so reinforcing their sense of self-esteem and accomplishment (Mendoza & Venables, 2023). This, in turn, serves as an additional incentive for them to actively participate in course materials and activities.

According to Sepehr and Head (2013), using competition as a game element to engage students in learning can be an effective strategy. Competition is important in gamification in educational settings due to its motivation for student engagement to fulfil learning objectives. According to Reeves and Read (2009), when students feel a sense of control in a gamification environment, they can be more actively engaged.

H4 *Competition (CP) has a positive influence on Student Engagement*.

In this study, we chose to focus on the examination of *Student Academic Achievement (SAA)* rather than pure academic performance. While students’ academic performance has always been recognised as a decisive outcome of student engagement and is often used to anticipate an educational system’s success, evaluate school performance, and provide a general overview of a student’s overall academic abilities and accomplishments (Zhao et al., 2005), SAA focuses specifically on the attainment of learning objectives and standards within individual subjects or courses and is often assessed qualitatively based on the demonstration of knowledge, skills, and competencies (Phelps, 2019). Since the context of our study is mainly based on a gamified hybrid course, it would be more reasonable to assess SAA rather than a broad overview of a student’s overall academic abilities and accomplishments of the whole study programme. In this sense, past studies have confirmed that enthusiasm, hard work, and actively engaging in learning activities can lead to success in student achievement and vice versa (Akbari et al., 2021). These claims indicate that perceived student engagement can positively enhance the academic achievement of students in gamified hybrid courses. Accordingly, the study proposes the following hypothesis:

H5 *Student Engagement has a positive influence on Student Academic Achievement*.

Finally, student engagement is an influential attribute in academic satisfaction in the hybrid learning context and is attained when ‘students are successful in the learning and are pleased with their experience’ (Moore, 2010). According to Wu et al. (2010), satisfaction refers to the aggregate of how a student feels and thinks, which is derived from combining all the beneficial aspects of a hybrid learning environment. Additionally, student satisfaction, as a momentary mindset deriving from an assessment of a student’s academic experience, is a positive antecedent of student loyalty and reflects the quality of an educational system (Zeithaml, 1988). Student engagement, which helps students to do their tasks, have better results, and know the lesson better, is claimed to positively influence student satisfaction (Akbari et al., 2021). Consequently, these factors indirectly improve student satisfaction regarding the process and the result. Hypothesis 6 (H6), therefore, is proposed as follows:

H6 *Student Engagement has a positive influence on Student Satisfaction (SS)*.

**Figure 4.**
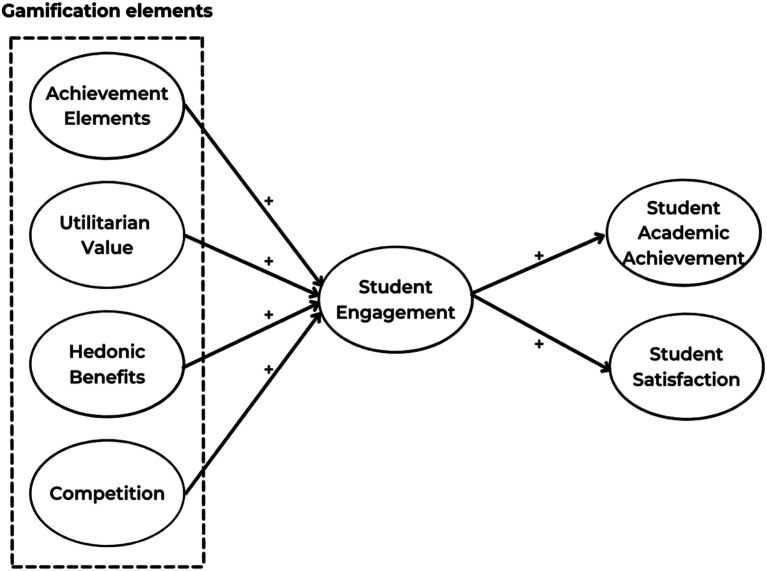
Research model.

With the combination of all the abovementioned factors, the proposed model is schematically presented in Figure 4.

## Methodology

### Research Design and Data Collection

The methodology we employ for this study consists of two distinct steps:

Initially, we utilised design science (DS) to include the technique of gamification into a hybrid course known as the ‘Global supply chain management’ which is a mandatory business course offered by the University. Throughout the three semesters of 2022, we implemented the new hybrid style of the course for about 2000 students.

Second, our objective is to conduct a quantitative empirical examination of the beneficial outcomes of incorporating gamification into the curriculum design for hybrid classrooms, utilising design science and analysing the complex mechanisms at play. Our study’s research object shall be the students who are participating in the gamified hybrid courses that followed our adopted DS framework and their study outcomes.

Specifically, a design science (DS) analytical framework, based on the seven core elements proposed by Thuan et al. (2019), was employed to develop a ‘roadmap’ for integrating gamification into university curriculum design. This study utilise design science research (DSR) to systematically design, synthesise, test, and evaluate educational artifacts – such as learning management systems (LMSs) and gamification strategies – that address practical educational challenges (Peffers et al., 2007). DSR’s focus on creating innovative, useful artifacts complements the behavioural sciences, which seek to uncover the underlying truths of phenomena (Hevner et al., 2004; Niederman & March, 2012). By combining these approaches, our research aims to advance the pedagogical field through the development and application of gamified educational tools.

**Figure 5.**
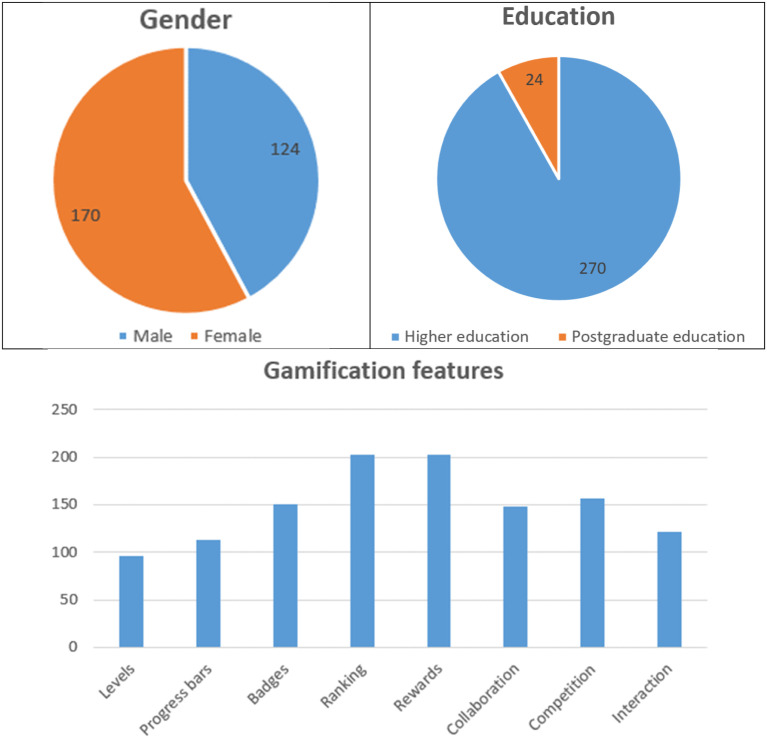
Profiling of respondents.

A subsequent survey was conducted to collect empirical data for this research. Measure items are provided in Appendix 1. The target population was the abovementioned students at the University from both undergraduate and postgraduate programs. This institution was selected for the survey due to its status as one of the pioneers in implementing hybrid learning in Ho Chi Minh City during the study period. Students at this university are tech-savvy and experienced in learning gamification. The survey consisted of two phases: a preliminary survey and an official survey. At the preliminary survey stage, the research team collected data from 50 people to check the scale’s reliability. The reliability test of the scale includes testing for Omni directionality, reliability, convergent value, and discriminant validity. If necessary, the scale will be adjusted accordingly. After completing the scale, the research team conducted an official survey. As a result, we received a total of 294 responses (91.8% undergraduate and 8.2% postgraduate due to the number of ongoing classes in the studied semester) based on a non-probability (3-semester quota) sampling approach, equivalent to a response rate of 59%. In our sample of research, the student’s gender is relatively balanced between the two sexes (42.2% and 57.8%). This helps to determine the factors that influence intentional behaviour across genders. Gamification features that affect students the most are ranking and rewards (202 and 203 votes), followed by Competition, Badges, and Collaboration, with a high selection of 157, 150, and 148, respectively. The remaining features are Interaction, Progress bars, and Levels, which also received fewer choices (122, 113, and 96, respectively), referring to Figure 5.

### Data Analysis

The data was then imported into Smart PLS software version 3.3.7 and the partial least squares structural equation modelling (PLS-SEM) method has opted for this empirical study, as the PLS-SEM method was recommended for the empirical research that aims to examine complex and intricate relationships or seeking theory testing developed on established theoretical foundations.

#### Assessment of the Indicator Reliability

The factor loading coefficient index is used to measure the reliability of the scale. Scales with factor loading coefficients less than 0.4 should be removed from the research model, whilst strong factor loadings ( ≥ 0.7) should be kept. For scales with factor loading coefficients from 0.4 to less than 0.7, the removal of the scale is only done if this removal increases the coefficients of the composite reliability coefficient or the coefficients of the mean extracted variance (Bagozzi et al., 1991; Hair et al., 2011).

After many runs of the ‘PLS Algorithm’ function in Smart PLS software, the scales in the model all have factor loading coefficients greater than 0.4 and strong, and only 3 scales, SE2, SS2, and UV2, are below 0.7. However, it does not affect the coefficients of the coefficient composite reliability or mean extracted variance coefficients; it should be kept in the model as all the scales are reliable.

#### Assessment of Discriminant Validity

Discriminant validity assessment is based on two indicators: Cross-loading and Fornell–Larcker (Hair Jr. et al., 2017).

With the cross-loading index, the observed variable must have the largest outer loading coefficients on the scale that the observed variable reflects compared to any other scale (Hair Jr. et al., 2017). In Table 1, all observed variables have outer loading coefficients with the scale that the observed variable reflects larger than all other scales. All scales reflect discriminant validity.

**Table 1.** Cross-Loading Coefficients.

	AM	CP	HB	SAA	SE	SS	UV
AM3	0.937	0.018	0.053	0.054	0.079	0.195	−0.123
AM4	0.815	−0.03	0.065	−0.015	0.047	0.188	−0.117
CP1	0.012	0.715	0.31	0.411	0.302	0.432	0.38
CP2	−0.029	0.593	0.226	0.323	0.301	0.237	0.21
CP3	0.004	0.787	0.294	0.47	0.454	0.387	0.411
CP4	−0.012	0.809	0.282	0.455	0.44	0.381	0.452
CP5	0.015	0.774	0.296	0.533	0.532	0.466	0.468
HB1	0.092	0.17	0.748	0.225	0.206	0.366	0.078
HB2	0.118	0.09	0.714	0.119	0.146	0.267	0.012
HB3	0.04	0.152	0.709	0.192	0.182	0.279	0.062
HB4	0.012	0.422	0.741	0.548	0.507	0.515	0.472
SAA1	0.036	0.554	0.455	0.822	0.596	0.556	0.501
SAA2	0.017	0.402	0.351	0.687	0.512	0.443	0.387
SAA3	0.001	0.397	0.371	0.752	0.539	0.509	0.432
SAA4	0.04	0.49	0.33	0.8	0.533	0.538	0.439
SE1	−0.011	0.425	0.329	0.552	0.742	0.436	0.522
SE2	0.029	0.33	0.24	0.451	0.674	0.411	0.383
SE3	0.067	0.468	0.369	0.562	0.786	0.542	0.457
SE4	0.126	0.445	0.415	0.534	0.752	0.587	0.443
SS1	0.039	0.399	0.425	0.487	0.451	0.729	0.407
SS2	0.247	0.342	0.389	0.438	0.451	0.686	0.351
SS3	0.237	0.474	0.479	0.552	0.571	0.816	0.423
SS4	0.137	0.39	0.43	0.572	0.587	0.84	0.435
UV1	−0.106	0.447	0.405	0.416	0.44	0.496	0.732
UV2	−0.112	0.177	0.08	0.248	0.288	0.216	0.517
UV3	−0.145	0.373	0.167	0.438	0.483	0.364	0.8
UV4	−0.035	0.466	0.305	0.493	0.492	0.384	0.755

When the indicator loadings were compared to other reflecting indicators in the cross-loading, discriminant validity was determined. Fornell and Larcker (1981) advocated using AVE with a score of 0.50 or more as a general guideline for assessing discriminant validity. For adequate discriminant validity, the square root of the AVE should be greater than the correlations among latent constructs (Fornell & Larcker, 1981). The Fornell–Larcker index compares the square root of a factor’s AVE with the correlation index of another factor. In particular, the square root of the AVE of a factor must be greater than the maximum correlation value with any other factor. In Table 2, the outcome from SmartPLS 3.3.7 shows that all the square root values of the AVE of the factors are larger than the correlation values of other factors.

**Table 2.** Fornell–Larcker Coefficients.

	AM	CP	HB	SAA	SE	SS	UV
AM	0.878						
CP	−0.000	0.74					
HB	0.065	0.38	0.728				
SAA	0.031	0.604	0.494	0.767			
SE	0.075	0.569	0.464	0.712	0.739		
SS	0.216	0.522	0.559	0.669	0.675	0.77	
UV	−0.135	0.535	0.352	0.576	0.612	0.525	0.709

#### Assessment of the structural model

Based on our analytical results, the assessed model does not have multicollinearity when the VIFs are less than 5 (Hair Jr. et al., 2017). Next, to examine the relevance of the path coefficients, this study uses the PLS standard bootstrapping process with 5000 bootstrap samples and 294 instances (Hair Jr. et al., 2014). The estimates for the entire structural model, which contains all variables, are shown in Table 3. Based on the *p*-value, we can confirm that all the hypotheses are accepted. The *p*-value, mainly used to determine significance, is interpreted as the probability of errors in a hypothesis rejection (Hair Jr. et al., 2017). With all *p*-values less than 5%, it can be concluded that the hypotheses in Table 3 are statistically significant.

**Table 3.** Structural Model Assessment Direct Relationship.

Hyp	Relation	Original sample (β)	Sample mean (M)	Standard deviation (STDEV)	T-value	*p* values	Findings
H1	AM -> SE	0.117	0.114	0.052	2.261	0.024	Supported
H2	UV -> SE	0.409	0.408	0.059	6.969	0.000	Supported
H3	HB -> SE	0.210	0.211	0.046	4.612	0.000	Supported
H4	CP -> SE	0.270	0.272	0.062	4.331	0.000	Supported
H5	SE -> SAA	0.712	0.715	0.037	19.196	0.000	Supported
H6	SE -> SS	0.675	0.677	0.040	16.985	0.000	Supported

Specifically, AM had a positive effect on SE (β = 0.117, t = 2.261, *p* < .05). The variable UV has a positive effect on SE, with the index of H2 being β = 0.409, t = 6.969, *p* < .05. Variables HB and CP also have similar results (β = 0.210, t = 4.612, *p* < .05 and β = 0.270, t = 4.331, *p* < .05). With H5, the value of β = 0.712, t = 19,196, *p* < .05 shows that SE has a positive influence on SAA, so the hypothesis is accepted. Finally, hypothesis H6 with variable SE has a positive effect with values of β = 0.675, t = 16,985, *p* < .05 supported this effect.

In Table 4, the adjusted R squared of the variable ‘Student Engagement’ is 0.504, so the independent variables explained 50.4% of the variation of the variable ‘Student Engagement’. The remaining 49.6 % is from systematic error and other factors outside the model. In addition, the R square adjusted for ‘Student Academic Achievement’ and ‘Student Satisfaction’ is 0.506 and 0.453, respectively, which means that the variable ‘Student Engagement’ explained 50.6% of the variation of the variable ‘Student Academic Achievement’ and 45.3% variation of the variable ‘Student Satisfaction'.

**Table 4.** *R*^2^ Value of Dependent Variables.

	R square	R square adjusted
Student Academic Achievement	0.507	0.506
Student Engagement	0.510	0.504
Student Satisfaction	0.455	0.453

## Discussion

### Impact of Gamification Elements on Student Engagement, Achievement, and Satisfaction

After successfully embedding gamification in curriculum design for the focal hybrid course (previously discussed in 4.1.), the study has positively applied the SOR theory to validate the impact of gamification elements (as stimuli), including achievement elements, utilitarian value, hedonic benefits, and competition, as well as the mediating impact of student engagement (organism) and student achievement and satisfaction (responses).

More specifically, our research shows that UV plays a key part in getting students to become more engaged (with the largest effect size of 0.409 among all gamification elements). Students were highly inspired by UV owing to its focus on efficiency and specific outcomes for each gamified activity. Not only did UV assist students, but it also additionally made learning appear worthwhile by offering them clear goals, relevant feedback, and explicit prizes. Consistent with earlier studies (Aguiar-Castillo et al., 2019; Baptista & Oliveira, 2019; Yang et al., 2021), our finding confirms that UV plays a crucial role in gamified learning settings in increasing the satisfaction and engagement of students in hybrid mode.

In addition, our study highlights how both hedonic benefits and competition elements could fairly boost student engagement and satisfaction (with smaller effect sizes of 0.210 and 0.270). Gamification features, such as stories, rankings, challenges, and incentives, make learning more engaging and immersive, which in turn increases student engagement in both online and in-person learning. This finding is consistent with previous research that students are more engaged and motivated in learning environments when there is some level of competition and hedonic benefits in gamified contexts (Reeves & Read, 2009; Sepehr & Head, 2013; Yusof et al., 2021). Achievement elements, such as badges, trophies, stars, levels, and progress bars, also bring significant impacts, despite the smallest effects on promoting student engagement empirically shown. Moreover, gamified achievement elements, when designed properly, may boost motivation and performance, as Groening and Binnewies (2019) confirmed.

Overall, our study presents empirical evidence that gamification components can improve hybrid course engagement, achievement, and satisfaction. HEI educators may develop more dynamic and immersive gamified learning experiences that promote active involvement, clear goals, and improved learning outcomes among students by incorporating gamification into the design of hybrid learning environments as proposed by our study (see Figures 1 and 2).

### Implications of the Study

In this study, the factors applied in gamification have an impact on the behaviour, learning outcomes, and satisfaction of students in higher education. Hence, student insights are drawn for educational administrators to implement appropriate training policies and curriculum design to increase student engagement, performance, and satisfaction. First, lecturers and instructors can convey useful information in familiar traditional forms, such as sentences and pictures, in a trending way to provoke interest from students, who are most sensitive to trends. In addition, learning through traditional books and reading materials can cause boredom, dizziness, and distraction. Therefore, applying more game elements will attract students’ interest in the subject. Additionally, lecturers and instructors can take advantage of the novelty of gamification to guide students to simplify knowledge from subject topics into real life because some knowledge is burdensome and hard to grasp. Lecturers and instructors can build a course-level roadmap with effective gamification features, including a progress bar, badges, ranking, rewards, and competition to provide an attractive learning progress overview and improve student engagement.

Another important factor that should be mentioned is competition. Competition with an impact coefficient of β = 0.270 is considered to have an important effect, after utilitarian value, on students’ active participation, which supports hypothesis H4. According to the result of the items’ outer weight in the variable, CP4: ‘Competition increases my participation in the game’ has the highest outer weight of 0.809. According to the World Economic Forum’s Global Competitiveness Report in the 4.0 period in 2019, Vietnam ranked 97th out of 141 countries in the ‘Skills’ pillar in the ‘Capability’ group. It can be said that the issue of competitiveness is one of the hardships for Vietnamese students in the 4.0-era labour market. Therefore, the research team put forward the second proposal: Universities and administrators need to approve the design of gamification to enhance competitiveness combined with organised lectures to create a competitive learning environment between students on campus and outside.

More specifically, in the classroom application, instructors can design games for groups or individuals with debate. Debates prompt opportunities for students to show opinion and bravery towards the opponent, as well as the satisfaction of winning over others that incites an autonomous feeling as the need to feel free and self-directed. Another way to increase the student experience in a competitive environment is to set goals for students' so-called extinction motivation. The competition gives a sense of competence which defines the need to feel effective when students achieve their goals.

### Limitations and Future Research

This research has a few shortcomings. The first limitation is that this study solely focused on students at the University instead of including all students in higher education in a hybrid learning environment in Ho Chi Minh City right after the COVID-19 epidemic. In future studies, researchers could expand the number of participants and vary the sampling process. Face-to-face interviews using a paper survey, going to places where the survey’s segmented people are aimed at, or appealing and useful presents for respondents are just a few examples. This would give more comprehensive responses and samples, allowing for more reliable research.

The lack of qualitative expert viewpoints is the study’s second shortcoming. Many HE curriculum development experts in this discipline were unable to participate in the survey due to time limits. Furthermore, because the study was conducted online, some responses were uncertain. As a result, many participants answered in a shallow and unfocused manner, considerably influencing the research findings. Furthermore, many schools use gamification in the classroom, particularly in the context of hybrid learning; hence, further research should be undertaken in different educational contexts, perhaps across various disciplinary areas, types of HEIs, regional areas, or cross-cultures to validate the generalisability of our framework. It can complement our study results and ensure variety and distinction in identifying the influential aspects of student behaviour.

The third limitation is related to the course’s learning roadmap, which is only a conceptual proposal to implement gamification in curriculum design based on DS. A practical framework with clear and up-to-date gamification-supported applications could be developed. Researchers can further investigate, propose, and validate an implementation process based on DS to assist educators in designing a highly engaging curriculum to improve student academic performance and satisfaction.

## Conclusion

Most of today’s students are digital natives, having grown up with digital technologies. Important issues referring to the adaptation of the learning process for students with diverse learning styles and new guidelines for teaching and learning must be resolved by lecturers and instructors. Especially post-COVID-19, it is critical to design innovative teaching tactics that increase students’ motivation and commitment while also maximising their knowledge acquisition. Among the many approaches, gamification has gained the attention of educators, who have been investigating its potential to increase student learning in recent years (Dichev & Dicheva, 2017; Koivisto & Hamari, 2019; Majuri et al., 2018). Gamification is an effective method for promoting positive changes in the behaviour and attitude of students toward learning, thereby increasing their motivation and engagement. In addition to influencing students’ performance and comprehension of pedagogical material, the change can also enable an effective learning process. This study has partly clarified why gamification elements (achievement elements, utilitarian value, hedonic benefits, and competition) applied to learning effect student engagement, performance, and satisfaction in the hybrid learning context. The study also examined the feasibility of incorporating gamification and design science into the teaching methodology. In this paper, we consider gamification an umbrella term for incorporating various games and other interactions to increase student engagement.

In summary, this study attempted to determine factors that improve student engagement, performance, and satisfaction via gamification elements in the hybrid learning context. Referring to a broad literature review of the Stimulus-Organism-Response model (SOR model), a comprehensive integration of cognitive and emotional states of individual variables affecting student engagement, performance, and satisfaction via gamification elements in a hybrid learning context has been proposed and confirmed. Based on the design science approach, a course-level learning roadmap was recommended to provide students with a gamification-based holistic view of the course learning progress. As a result, it would improve student engagement, academic achievement, and satisfaction.

## Data Availability

The data are available on request from the corresponding author due to the participant privacy.

## APPENDIX

### Appendix 1. Measure Items

Measure items are based on past studies in the field of gamification/education and modified to match the context of the Vietnam education system and hybrid learning context.

**Table A1.** Item scale for all constructs.

Constructs	Items	Sources
Achievement Elements	AM1: I was able to track my achievement progress via gamification.	Kankanhalli et al. (2005)
	AM2: I was able to remember the knowledge I gained from the gamified quiz work	
	AM3: Awards increase my involvement in the game.	
	AM4: I try to get more points as a reward for my activities.	
	AM5: I try to get more badges or loot as a reward for my activities.	
Utilitarian Value	UM1: I think gamification is useful to study the subject.	Bilgihan and Bujisic (2015)
	UM2: I think it is interesting about the information which gamification gives me.	
	UM3: I think the information about the subject is useful to know it deeply.	
	UM4: I find gamification useful to improve my mark.	
Hedonics Benefits	HB1: Playing gamification in class can be relaxing.	Bilgihan and Bujisic (2015)
	HB2: Playing gamification in class is enjoyable.	
	HB3: I thought that this class was fun because of the gamified quiz assessments.	
	HB4: I felt enthusiastic to participate in a gamified learning activity.	
Competition	CP1: When I'm playing gamification I want to do better than other players.	Tomaselli et al. (2015)
	CP2: When I'm playing gamification my goal is to get better scores or rewards than other players.	
	CP3: Playing gamification allows me to be competitive.	
	CP4: Competition increases my participation in the game.	
	CP5: Playing gamification allows me to compare my skills with those of other players.	
Student Engagement	SE1: I frequently interacted with my instructor.	Dunn and Kennedy (2019)
	SE2: I participated in synchronous and/or asynchronous chat sessions during the class.	
	SE3: I have meaningful interactions with other students while using gamification.	
	SE4: I am personally interested in the topics I learn about while using gamification.	
Student Academic Achievement	SAA1: I am confident I will do well on tests.	Bryan et al. (2011)
	SAA2: I believe I can earn a high grade.	
	SAA3: I prepare well for course tests and assignments.	
	SAA4: I believe I can master my course's knowledge and skills.	
Student Satisfaction	SS1: I am satisfied with my overall experience in this gamification.	Gray and DiLoreto (2016)
	SS2: I am satisfied with my learning in the class with gamification.	
	SS3: I am satisfied with the level of student interaction that occurred in the course.	
	SS4: I am satisfied with the content of the lesson.	
